# Serum Starvation-Induced Voltage-Gated Potassium Channel Kv7.5 Expression and Its Regulation by Sp1 in Canine Osteosarcoma Cells

**DOI:** 10.3390/ijms15010977

**Published:** 2014-01-10

**Authors:** Bo Hyung Lee, Pan Dong Ryu, So Yeong Lee

**Affiliations:** Laboratory of Veterinary Pharmacology, College of Veterinary Medicine and Research Institute for Veterinary Science, Seoul National University, 1 Gwanak-ro, Gwanak-gu, Seoul 151-742, Korea; E-Mails: bohyung2@snu.ac.kr (B.H.L.); pdryu@snu.ac.kr (P.D.R.)

**Keywords:** *KCNQ5*, CCL-183, flupirtine, voltage-gated potassium channels, cell cycle arrest, proliferation

## Abstract

The *KCNQ* gene family, whose members encode Kv7 channels, belongs to the voltage-gated potassium (Kv) channel group. The roles of this gene family have been widely investigated in nerve and muscle cells. In the present study, we investigated several characteristics of Kv7.5, which is strongly expressed in the canine osteosarcoma cell line, CCL-183. Serum starvation upregulated Kv7.5 expression, and the Kv7 channel opener, flupirtine, attenuated cell proliferation by arresting cells in the G_0_/G_1_ phase. We also showed that Kv7.5 knockdown helps CCL-183 cells to proliferate. In an effort to find an endogenous regulator of Kv7.5, we used mithramycin A to reduce the level of the transcription factor Sp1, and it strongly inhibited the induction of Kv7.5 in CCL-183 cells. These results suggest that the activation of Kv7.5 by flupirtine may exert an anti-proliferative effect in canine osteosarcoma. Therefore, Kv7.5 is a possible molecular target for canine osteosarcoma therapy.

## 1. Introduction

Voltage-gated potassium (Kv) channels are involved in the regulation of cell excitability and are one of the most diverse ion channel families. In addition to playing an important role in excitable cells, Kv channels also exist in non-excitable cells, such as alveolar epithelial cells [1] and immune cells [2,3]. Moreover, recent studies have demonstrated the involvement of several Kv channels in cancer cell proliferation [4–12].

M-currents were first discovered in 1980 as non-inactivating channels that slowly activate and deactivate [13]. Later, it was found that Kv7 channels contribute to M-currents and that mutations in the *KCNQ* genes lead to hereditary channelopathies, such as benign familial neonatal convulsions [14], long QT syndromes, epilepsy, and congenital deafness [15]. Kv7.1, which is encoded by *KCNQ1*, predominantly exists in cardiac cells; four other subunits from Kv7.2 to Kv7.5, each encoded by *KCNQ2* to *KCNQ5*, respectively, are present in the brain and primary sensory cells [16]. Kv7 is expressed in non-neuronal tissues as well, including skeletal muscle, myoblasts [17], and various smooth muscles, such as the digestive system [18], the airway [19], and the bladder [20] tissues. Recent studies have shown that the Kv7 family is involved in other cell regulation processes, such as cell signaling [21], cell proliferation, and differentiation [17,22].

The ubiquitously expressed transcription factor specificity protein 1 (Sp1) was the first transcription factor to be cloned from mammalian cells in 1983 [23], and its role has been investigated in various cells. Sp1 binds to GC-rich regions in the promoters of genes and activates transcription [24]. It is also known to physically interact with other transcription factors to regulate thousands of genes associated with various cellular processes, such as cell proliferation, apoptosis, and tumorigenesis [25].

In the current study, for the first time, we identified strong expression of Kv7.5 in cancer cells and examined the hypothesis that Kv7.5 is involved in the regulation of cancer cells and serve as a possible therapeutic target in canine osteosarcoma.

## 2. Results

### 2.1. Identification of Kv7.5 in the CCL-183 Canine Osteosarcoma Cell Line

**Figure 1.**
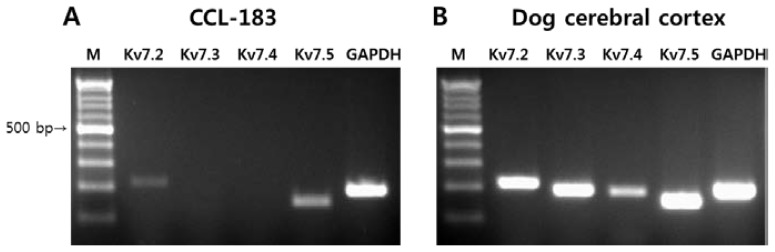
RT-PCR analysis of the *KCNQ* gene family. PCR products using cDNA from the CCL-183 cell line (**A**) and dog cerebral cortex (**B**) were electrophoresed on a 2% agarose gel. M, DNA ladder.

The mRNA expression of the *KCNQ* gene family including Kv7.2, Kv7.3, Kv7.4, and Kv7.5 in the CCL-183 cells was analyzed using RT-PCR (Figure 1A). Dog cerebral cortex was used as a positive control, and we confirmed the appropriate sizes for Kv7.2, Kv7.3, Kv7.4, and Kv7.5 (Figure 1B). As shown in Figure 1A, Kv7.5 was the most highly expressed Kv7 channel in the CCL-183 cells. Therefore, we chose Kv7.5 for subsequent experiments.

### 2.2. Serum Starvation Upregulates Kv7.5 Transcripts and Protein in a Time-Dependent Manner

**Figure 2.**
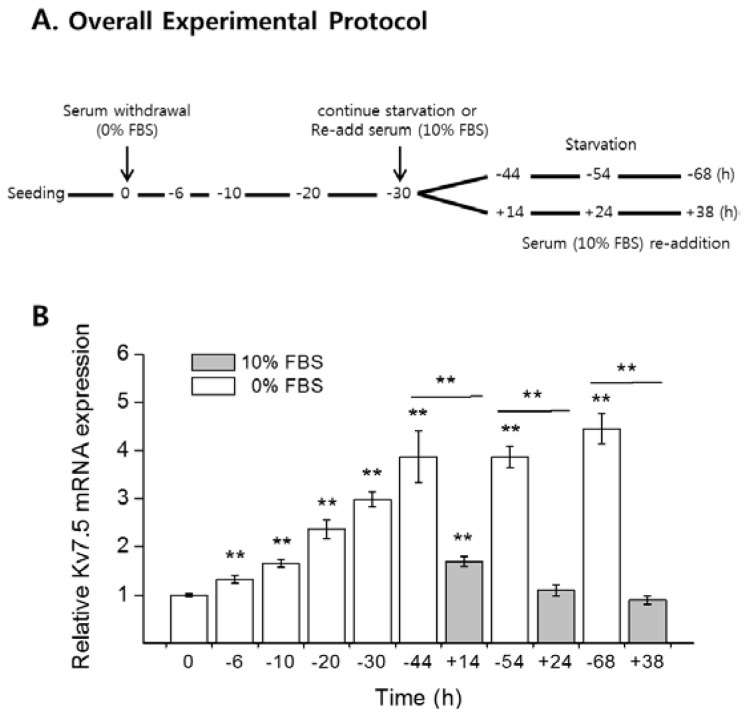

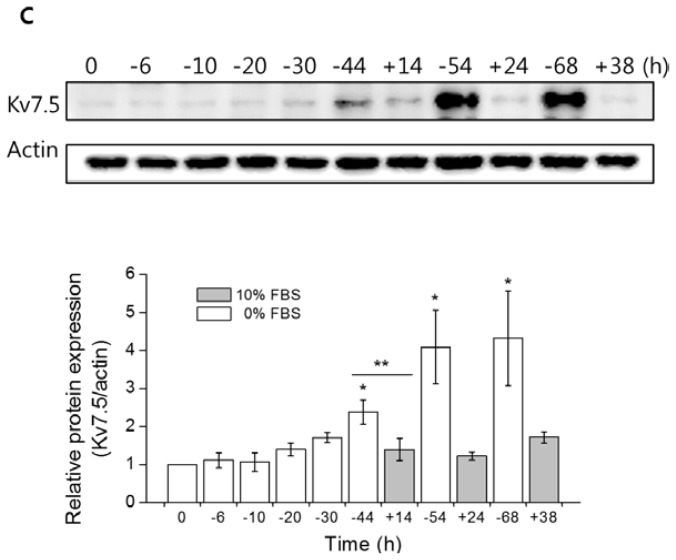
Upregulation of Kv7.5 expression levels by serum deprivation. (**A**) The cells were seeded onto plates and incubated overnight before serum withdrawal. On the following day, one plate of cells was harvested as a control for the experiments (0 h), and the other subconfluent proliferating cells were washed two times with warm PBS and transferred into serum-free DMEM. The cells were further incubated for 6, 10, 20, 30, 44, 54, and 68 h (prefixed with “−” to imply the withdrawal of serum) and harvested. Three plates of cells were transferred into complete growth medium after 30 h of serum deprivation to induce the cell progression into the G_1_–S transition and incubated for 14, 24, and 38 h (prefixed with “+” to imply the re-addition of serum); The relative expression levels of Kv7.5 in the presence or absence of FBS were analyzed by qPCR (**B**) and western blot analysis (**C**). The values are the mean ± SEM of five (**B**) and four (**C**) independent experiments. The asterisks denote values significantly different from the control (0 h). * *p* < 0.05; ** *p* < 0.01.

To examine the effect of serum starvation on Kv7.5 expression in CCL-183 cells, subconfluent proliferating CCL-183 cells were serum starved for up to 68 h (0, −6, −10, −20, −30, −44, −54, and −68 h), and serum was re-added at −30 h. The cells were then allowed to incubate until three different time points (+14, +24, and +38 h) (Figure 2A). Serum-deprived cells accumulated in the G_0_/G_1_ stage in a time-dependent manner, and cells re-exposed to serum progressed through the G_1_–S transition, recovering their normal proliferation state (0 h) (Figure S1).

The cells that were harvested at the indicated times after serum starvation (0% FBS) and re-addition (10% FBS) were analyzed with qPCR to observe changes in Kv7.5 mRNA levels. Figure 2B shows that the Kv7.5 mRNA level was significantly increased up to 4.5 times from 0 (1.00 ± 0.03) to 68 h (4.45 ± 0.32) in a time-dependent manner, and when cell proliferation was triggered by serum re-addition, the Kv7.5 level was significantly decreased relative to the control level (0 h). We also examined the changes in Kv7.5 expression at the protein level; Figure 2C shows that the protein changes corresponded to the changes in the mRNA. It demonstrates that the protein level of Kv7.5 was significantly increased up to approximately 4.3 times (4.32 ± 1.24) compared to 0 h when the cells were serum starved for 68 h.

### 2.3. Involvement of Kv7.5 in CCL-183 Cell Proliferation

**Figure 3.**
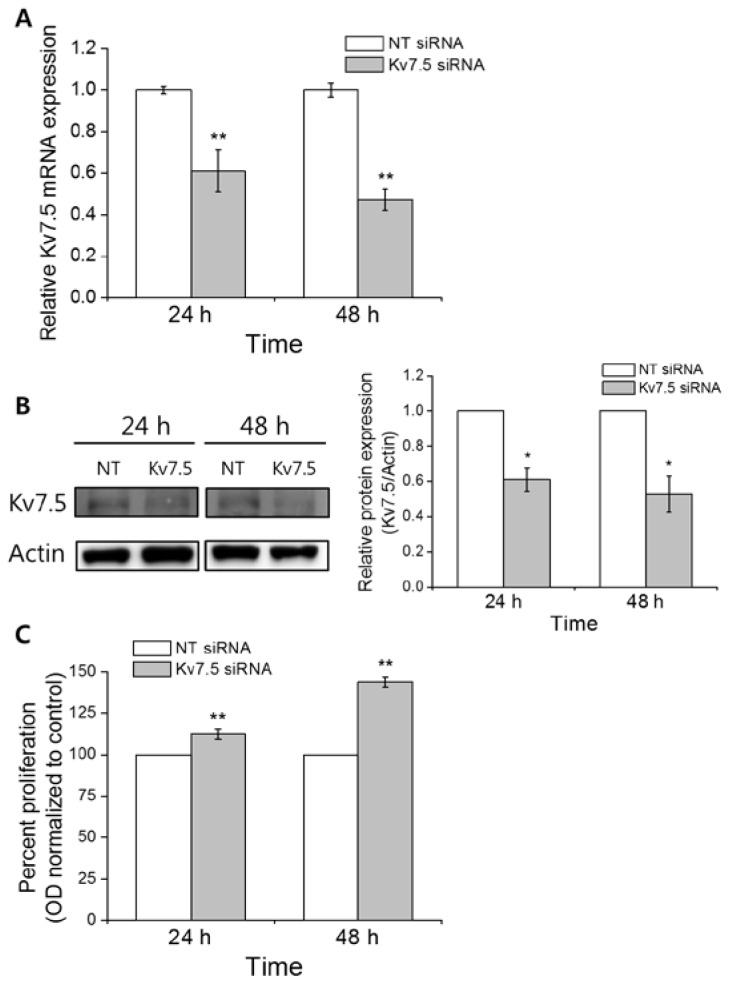
Kv7.5 knockdown by siRNA transfection induces proliferation of CCL-183 cells. The effect of transient knockdown of Kv7.5 in Kv7.5 mRNA (**A**) and protein (**B**) expression in CCL-183 cells was analyzed. The values are the mean ± SEM of four independent transfections; (**C**) An MTT cell proliferation assay was performed on the cells transfected with siRNA, and increased proliferation in Kv7.5-knockdown cells was observed compared to the cells transfected with NT siRNA. The values are the mean ± SEM of 11 independent MTT assays. * *p* < 0.05; ** *p* < 0.01.

The upregulation of Kv7.5 at both the mRNA and protein levels in the cell cycle-arrested cells, as well as its decline in serum-stimulated proliferating cells, suggests a possible role for Kv7.5 in cell proliferation. To investigate the relationship between Kv7.5 and CCL-183 proliferation, we generated a transient knockdown of Kv7.5 in CCL-183 cells by transfection with siRNA against Kv7.5. Figure 3A shows the suppressed mRNA expression of Kv7.5 in these cells to 61% (24 h) and 47% (48 h) of its level in the NT siRNA-transfected cells. A western blot analysis also demonstrated decreased expression of the Kv7.5 protein in Kv7.5 siRNA-transfected cells to 61% (0.61 ± 0.07, 24 h) and 53% (0.53 ± 0.10, 48 h) of its level in the NT siRNA transfected cells (Figure 3B). The MTT assay performed on the siRNA-transfected cells revealed that cell proliferation was significantly increased by 12% (112 ± 0.03; 24 h) and 44% (144 ± 0.1; 48 h) compared with NT siRNA (Figure 3C).

### 2.4. Flupirtine, a Kv7 Opener, Arrests Cells in the G_0_/G_1_ Phase

**Figure 4.**
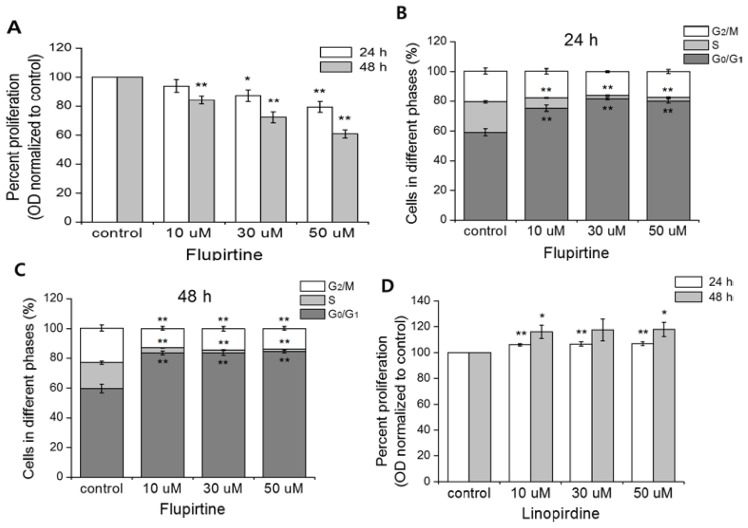
Changes in the cell cycle phase distribution and decreases in cell proliferation due to the Kv7 channel opener, flupirtine. (**A**) Flupirtine treatment (10, 30, and 50 μM) induced a decrease in the proliferation rate compared to the control in a concentration- and time-dependent manner. The values are the mean ± SEM of seven independent MTT assays. The asterisks denote values significantly different from each control (24 or 48 h). The treatment (with flupirtine for 24 (**B**) and 48 h (**C**) demonstrated the inhibition of the G_1_–S transition in a concentration- and time-dependent manner. The values are the mean ± SEM of four independent flow cytometry assays. The asterisks denote values significantly different from each control G_0_/G_1_, S, or G_2_/M). (**D**) Linopirdine treatment (10, 30, and 50 μM) increased the proliferation rate compared to the control in a time-dependent manner. The values are the mean ± SEM of eight independent MTT assays. The asterisks denote values significantly different from each control (24 or 48 h). * *p* < 0.05; ** *p* < 0.01.

Next, we examined the effect of the Kv7 channel opener, flupirtine, on cell proliferation and cell cycle phase distribution. Figure 4A shows that flupirtine hinders cell proliferation in a time- and concentration-dependent manner. When incubated with 50 μM flupirtine for 48 h, proliferation of CCL-183 cells was significantly reduced by 39%. To evaluate the changes in the cell cycle while proliferation was arrested, we investigated the effect of flupirtine on cell cycle phase distribution using flow cytometry. We applied flupirtine to CCL-183 for 24 (Figure 4B) and 48 h (Figure 4C). Flupirtine has a profound effect on cell cycle arrest at each concentration (10, 30, and 50 μM) tested because the G_0_/G_1_ phase increased significantly from 59% to more than 80% on average (for 24 and 48 h), whereas the S phase declined noticeably by more than 13% on average (for 24 and 48 h). Together, these results reveal that flupirtine, a Kv7 channel opener, suppresses CCL-183 proliferation by interfering with the G_1_–S transition. To support this data, we used linopirdine, a Kv7 channel blocker that successfully inhibited the Kv7.5 current [26], to determine its ability to reverse cell proliferation. Figure 4D shows increased cell proliferation when linopirdine (10, 30, and 50 μM) is applied. We did not observe a concentration-dependent increase, but the proliferation rate increased from an average of 107% (106% ± 1% with 10 uM; 107% ± 2% with 30 uM; 107% ± 1% with 50 uM; 24 h) to 117% (116% ± 5% with 10 uM; 118% ± 9% with 30 uM; 118% ± 5% with 50 uM; 48 h), indicating the time-dependent effect of linopirdine.

### 2.5. Mithramycin a Blocks Kv7.5 Transcription by Inhibiting Sp1 Binding to the Kv7.5 Promoter

To elucidate the transcription factors that may be responsible for driving endogenous *KCNQ5* expression, we used web-based prediction programs to analyze the possible transcription factors that bind to the *KCNQ5* promoter. We used the following software: TFSEARCH (http://www.cbrc.jp/research/db/TFSEARCH.html) and TFBIND (http://tfbind.hgc.jp/). The promoter region of the dog *KCNQ5* gene was analyzed, and both software programs predicted that the region consisting of −1114 to −1005 bp (XM_532200.3 was used to set the transcription start site as +1) had a GC-rich box with a high probability score for Sp1 binding.

**Figure 5.**
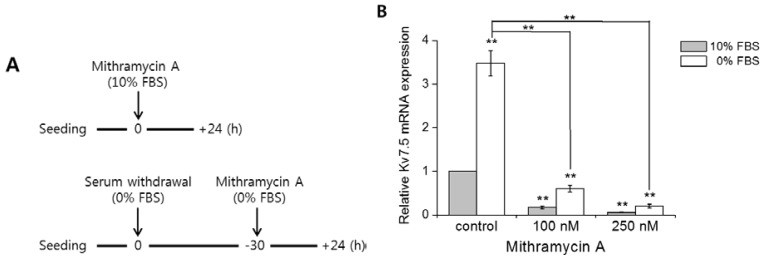

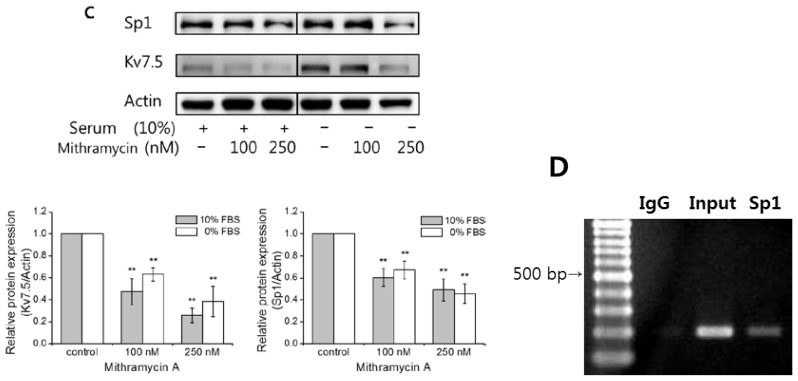
Inhibition of Sp1 by mithramycin A reduces Kv7.5 expression. (**A**) The cells were treated with mithramycin A for 24 h in serum-supplemented (10% FBS) or in serum-deprived (0% FBS) medium. The cells were seeded in plates in complete growth medium and incubated overnight. Three of the plates were treated with mithramycin A (100 and 250 nM) and a vehicle (control) in complete growth medium (top), and the other plates were washed twice with warm PBS and incubated for 30 h in serum-free medium (0% FBS). After 30 h of serum starvation, the plates were treated with mithramycin A (100 and 250 nM) and a vehicle (control) in serum-free medium (bottom); (**B**) The relative mRNA expression levels of Kv7.5 in the control and mithramycin A-treated cultures with serum (10% FBS) or without serum (0% FBS) were measured by qPCR and normalized against GAPDH expression. The values are the mean ± SEM of six independent experiments. The asterisks denote values significantly different from the control (10% FBS), ** *p* < 0.01; (**C**) The changes in Kv7.5 at the protein level after mithramycin A treatment for 24 h in serum-supplied or serum-deprived cells were analyzed by western blot analysis. Protein quantification was performed with four separate determinations, and the values are the means ± SEM. The asterisks denote values significantly different from each control (10% or 0% FBS). ** *p* < 0.01; (**D**) A ChIP assay was performed with the antibodies against Sp1 or nonspecific IgG. The Kv7.5 promoter region was expected to have a GC box for Sp1 binding and was amplified by RT-PCR. The final products were run on a 2% agarose gel to confirm the appropriate size (194 bp).

To determine whether Sp1 plays a role in Kv7.5 transcription, we used mithramycin A, which hinders the binding of Sp1 to the GC-rich promoter region [24,27] and inhibits the function of Sp1 [28]. Because serum starvation elevates Kv7.5 mRNA and protein expression (Figure 2B,C), two types of experiments were designed. Figure 5A demonstrates the overall protocol. The top panel shows the incubation of cells with mithramycin A in complete growth medium (10% FBS), while the bottom panel shows cells treated with mithramycin A in medium without serum (0% FBS) after 30 h of serum deprivation. The samples were harvested after 24 h of mithramycin. A incubation and analyzed by qPCR and western blot assays.

Figure 5B shows significantly decreased relative Kv7.5 mRNA expression both in serum-added and serum-starved cells in the presence of mithramycin A. The mRNA levels were reduced by 82% (100 nM of mithramycin A) and 94% (250 nM of mithramycin A) on average (for 10% and 0% FBS). Figure 5C shows the effect of mithramycin A on Kv7.5 protein expression, as determined by western blot analysis. The Kv7.5 level was strongly increased by serum starvation but decreased in both serum-added and serum-starved cells following mithramycin A treatment. It shows that the protein level of Kv7.5 and Sp1 was decreased to 55% and 64%, respectively (100 nM of mithramycin A), and 32% and 47%, respectively (250 nM of mithramycin A), on average (for 10% and 0% FBS).

Because this result revealed that the Kv7.5 gene is regulated by transcription factor Sp1, we then performed a ChIP assay to confirm the binding of Sp1 to the *KCNQ5* promoter. We designed primers that amplify the promoter-containing region (−1114 to −1005 bp) with a 194 bp PCR product. In this region, there were no other putative Sp1 binding sites. Figure 5D shows that Sp1 binds to this promoter region, as the input DNA (diluted 1:10) used as a positive control showed the existence of DNA, but anti-IgG failed to bind.

### 2.6. Serum Dependent Cell Cycle Progression

Cells harvested at the indicated time points described in Figure 2A were analyzed by a flow cytometry assay to observe changes in the cell-cycle phase distribution.

Figure S1A shows histograms of cells arresting in the G_0_/G_1_ stage (2C DNA content) following serum starvation and cells restoring to the normal proliferation state when 10% FBS was re-added. Figure S1B shows the cell count from the flow cytometry assay in a percentage distribution, and it shows a time-dependent increase in the cell distribution in the G_0_/G_1_ phase from 44% ± 2% (0 h) to 60% ± 6% (−68 h) when serum starved. The cells transferred into serum-supplemented medium re-entered cell progression, which indicated a significant increase in the S phase (between 2C and 4C DNA content) and a decline in the G_0_/G_1_ phase compared to serum-starved cells. The G_2_/M phase (4C DNA content) was maintained at a constant percentage (approximately 39% ± 4%) throughout the experiments (data not shown). The cells incubated for more than 24 h with 10% FBS, however, showed a decrease in the S phase because the cells reached maximum confluency.

## 3. Discussion

Here, we first identified Kv7.5 in canine osteosarcoma CCL-183 cells and demonstrated their anti-proliferative activity using flupirtine, a Kv7 channel opener, and siRNA-targeted Kv7.5. In addition, we demonstrated that the Kv7.5 mRNA transcript was upregulated by serum deprivation. Previously, Kv7.5 was proven to be upregulated in serum-induced proliferative myoblast cells [17]. In that study, myoblasts were serum deprived for 36 h to halt cell growth, and then serum was re-added, which led to a more than four-fold increase in the level of Kv7.5 mRNA expression. This result contrasted with our results, where serum withdrawal induced the upregulation of Kv7.5 transcripts in CCL-183 cells and serum re-addition helped the cells to restore proliferation with a low level of Kv7.5 transcripts. This discrepancy could have occurred due to the difference in cell types, in that myoblasts are normal cells compared to the CCL-183 osteosarcoma cells, which are derived from a malignant cancer, suggesting that the activation of Kv7.5 is cell-type specific.

Flupirtine has been used as an analgesic since 1984. It is neither an opioid nor a nonsteroidal anti-inflammatory drug used to treat cancer-associated neuropathic pain [29] and has the potential to be particularly effective in the control of pain arising from musculoskeletal tissues [30]. Its cytoprotective activity in cell cultures has been investigated, and flupirtine has been proven to counteract apoptosis when used at concentrations of 100 μM [31,32]. According to our results, the activation of Kv7.5 by flupirtine modulates cell proliferation, and whether this effect exists in other cancer cells needs to be investigated in the future. Another Kv7 activator, retigabine, reduced murine C2C12 myoblast proliferation, which is mainly mediated by Kv7.4, suggesting that Kv7 is a promising pharmacological target for regulating skeletal muscle proliferation [22].

Serum starvation is one of the methods for arresting cells in the G_0_/G_1_ phases that results in the transcriptional repression of several cell cycle regulatory genes [33]. In the present study, we discovered that serum deprivation induced the upregulation of Kv7.5, and its anti-proliferative effect was demonstrated.

The proliferative role of Kv channels was previously elucidated [34]. For example, the Eag1 channel (Kv10.1) helped human colonic carcinoma cells proliferate by affecting intracellular pH and Ca^2+^ signaling [35]. In human lung adenocarcinoma, selective blocking of Kv1.3 and the knockdown of Kv1.3 showed an anti-proliferative effect by affecting the G_1_–S transition, hence suggesting a role for Kv1.3 in cancer cell proliferation [36]. A hERG channel (Kv11.1) has been studied as a biomarker of cancer cells because various cancer cell lines exhibit significant amounts of hERG, whereas the corresponding normal cell lines do not [37]. Reduced expression of the hERG protein by siRNA transfection resulted in decreased cell proliferation in small cell lung cancer cells [38], and a hERG channel promoted the proliferation of ovarian cancer cells by affecting the cell cycle [39]. Interestingly, in the human brain tumor cells neuroblastomas and astrocytomas, the K^+^ channel opener, cromakalim, displayed antitumor activity via the activation of ATP-sensitive K^+^ channels, causing growth inhibition [40]. Similarly, we found that flupirtine, a Kv7 channel opener, exhibits an anti-proliferative effect in canine osteosarcoma cells. Therefore, Kv channels seem to have both proliferative and anti-proliferative roles, depending on the Kv channel subtype.

The transcriptional regulation of Kv7 is becoming better understood. Kv7.4 was shown to be downregulated via repressor element-1 silencing transcription factor (REST) [41], and Kv7.2 and Kv7.3 have been shown to be activated by Sp1 and repressed by REST [42]. In this study, we evaluated how transcription factor Sp1 could play an important role in driving Kv7.5 expression. Recent studies have shown that gene expression could be activated by an increase in Sp1 abundance in serum-deprived cells [43,44]. Serum-deprived CCL-183 cells showed elevated expression of Kv7.5; however, the increased level of Kv7.5 was reversed by treatment with mithramycin A, suggesting that Sp1 plays a critical role in regulating Kv7.5 expression.

Studies have also revealed that Sp1 plays a role in cell cycle arrest during the G_0_/G_1_ phase in human prostate cancer cells [45] and human breast cancer cells [46]. Similarly, we demonstrated that enhancing Kv7.5 expression using flupirtine promotes the arrest of CCL-183 cells in the G_0_/G_1_ stage. We also performed a ChIP assay to elucidate the putative Sp1 binding sites. Among the highly putative Sp1 binding sites, we confirmed that the region −1114 to −1105 bp upstream of *KCNQ5* is responsible for Sp1 binding. Taken together, these data suggest that the interaction of Sp1 with the GC box may be important for the expression of the endogenous *KCNQ5* in CCL-183 cells.

## 4. Experimental Section

### 4.1. Cell Culture

The canine osteosarcoma cell line CCL-183 was obtained from the American Type Culture Collection (Rockville, MD, USA). The cells were cultured in Dulbecco’s modified Eagle’s medium (DMEM) (Welgene, Daegu, Korea) supplemented with 10% (*v*/*v*) fetal bovine serum (FBS) (Welgene, Daegu, Korea), antibiotics, and an antimycotic solution (antibiotics): 10 U/mL penicillin, 10 μg/mL streptomycin, and 25 ng/mL amphotericin B (Sigma Aldrich, St. Louis, MO, USA). The cells were maintained at 37 °C in a 5% CO_2_-air-humidified atmosphere. In all experimental settings, the cells were seeded and maintained in this condition, unless otherwise stated.

To obtain cells arrested in the G_0_/G_1_ phase, cells seeded in complete growth medium (DMEM supplemented with 10% FBS and antibiotics) were incubated overnight and washed two times with warm (37 °C) phosphate buffered saline (PBS). The cells were then transferred into serum-free DMEM for the indicated time. After 30 h of serum deprivation, some G_0_/G_1_-arrested cells were then induced to re-enter the cell cycle by transferring them to complete growth medium and maintained in the indicated environment for the indicated time.

### 4.2. RNA Isolation: Reverse Transcription PCR (RT-PCR)

Treated cells were harvested by trypsinization, washed with ice-cold PBS, and pelleted. Total RNA was extracted using Trizol reagent (Takara Bio, Otsu, Japan) and the RNeasy Micro Kit (Qiagen, Hilden, Germany). Total RNA from dog cerebral cortex (Zyagen, San Diego, CA, USA) was treated with DNase I (Takara, Otsu, Japan), and the purity and concentration of the RNA were measured using a NanoDrop^®^ ND-1000 spectrophotometer (Thermo Fisher Scientific Inc., Boston, MA, USA).

cDNA was synthesized from 1 μg of total RNA using random primers and the M-MLV reverse transcriptase kit (Life Technologies, Paisley, UK), according to the manufacturer’s instructions. PCR controls were performed in the absence of reverse transcriptase (−RT). The PCR conditions involved an initial denaturation at 94 °C for 5 min, cycling (30 cycles) at 94 °C for 40 s, 55 °C for 40 s, and 72 °C for 1 min, with a final extension at 72 °C for 7 min. The final RT-PCR products were electrophoresed on a 2% agarose TAE gel stained with RedSafeTM nucleic acid staining solution (iNtRON Biotechnology Inc., Sungnam, Korea). The controls (−RT) were run on the same agarose gel, and it was confirmed that there was no contamination (data not shown). A DNA ladder (Elpis Bio, Daejeon, Korea) was used to confirm the PCR product size.

### 4.3. Quantitative Real-Time PCR (qPCR)

Quantitative real-time PCR was performed on a Step One Real-Time PCR System using SYBR Green I (SYBR Premix Ex Taq) (Takara, Otsu, Japan) and analyzed according to the manufacturer’s instructions (Applied Biosystems, Foster City, CA, USA). The PCR reactions were carried out under the following conditions: 95 °C for 30 s as an initial denaturation step and 95 °C for 5 s and 55 °C for 30 s as the PCR reaction step, which was repeated 40 times. Melting-cure analysis was performed immediately after the PCR reactions to confirm the absence of nonspecific PCR amplifications under the following cycling conditions: 95 °C for 15 s, 60 °C for 1 min, and 95 °C for 15 s. The comparative *C*_T_ method was used to quantify the expression of the target gene. The relative fold change of mRNA was calculated using the delta-delta *C*_T_ method (2^−ΔΔ^*^C^*^_T_^), normalized by the endogenous reference gene, glyceraldehyde 3-phosphate dehydrogenase (GAPDH). The primer sequences and accession numbers used for the RT-PCR and real-time PCR are presented in Table 1.

### 4.4. Small Interfering RNA (siRNA) Transfection

The cells were seeded into a 24-well plate one day before the transfection and incubated until they reached optimal confluency for transfection. They were then washed with warm PBS and transferred into 400 μL of antibiotic-free DMEM supplemented with 10% FBS. The cells were transfected by the addition of small interference RNA (siRNA) duplexes (Bioneer, Daejeon, Korea) at a final concentration of 70 nM with Lipofectamine 2000 (Life Technologies, Paisley, UK), following the manufacturer’s instructions. The siRNA duplex used to downregulate *KCNQ5* expression was designed by Bioneer, and the targeting sequences were forward, 5′-GACUUGGGCAAAUCUCUGUTT-3′ and forward, 5′-GUGAACAGACAUCUGACUATT-3′. Non-targeting siRNA (NT siRNA) was used as a negative control, as it did not target any known mammalian gene; the sequence was forward, 5′-AAUUCUCCGAACGUGUCACGU-3′ [47].

### 4.5. Drugs and Antibodies

Flupirtine (*N*-(2-Amino-6-(((4-fluorophenyl)methyl)amino)-3-pyridinyl) carbamic acid ethyl ester maleate) and linopirdine (1,3-dihydro-1-phenyl-3,3-bis(4-pyridinylmethyl)-2H-indol-2-one) from Tocris Bioscience (Bristol, UK) were dissolved in dimethyl sulfoxide (DMSO) (Sigma Aldrich, St. Louis, MO, USA) and water, respectively, to make 10 mM stock solutions, aliquoted, and stored in a frozen state at −70 °C. Mithramycin A (Sigma Aldrich, St. Louis, MO, USA) was dissolved in methanol (Sigma Aldrich, St. Louis, MO, USA) to make a 1 mM stock solution, which was stored at −20 °C in small aliquots. The drugs were diluted with the culture medium to achieve the desired concentrations immediately before use. In all experiments, the samples were compared to the vehicle control containing the same amount of solvent used in the samples. Propidium iodide (3,8-diamino-5-(3-diethylmethylamino)propyl)-6-phenyl phenanthridinium diiodide) (Sigma Aldrich, St. Louis, MO, USA) was dissolved in water at 1 mg/mL and stored in the dark at 4 °C. The colorimetric thiazolyl blue tetrazolium bromide (MTT) (Sigma Aldrich, St. Louis, MO, USA) was dissolved in cold PBS (50 mg/mL) immediately before use.

The Sp1 (39058) antibody was purchased from Active Motif (Carlsbad, CA, USA), and the Kv7.5 antibody (APC-155) was purchased from Alomone Labs (Jerusalem, Israel). Normal rabbit IgG (SC-2027) and actin (SC-1616 HRP) antibodies were purchased from Santa Cruz Biotechnology Inc. (Santa Cruz, CA, USA). Horseradish peroxidase (HRP)-conjugated goat anti-rabbit IgG (SA002-500) was obtained from GenDEPOT (Barker, TX, USA) and used as a secondary antibody.

### 4.6. MTT Cell Proliferation Assay

Cell proliferation was assessed by the MTT assay. The cells were seeded into a 24-well plate and incubated overnight. Cells were transfected with siRNA in the manner mentioned above or treated with drugs. Briefly, the cells were washed with PBS and transferred to media containing drugs of various concentrations (10, 30, and 50 μM). To normalize the effect of the DMSO or water used as the solvent, each experiment had a vehicle control that was exposed to the same amount of solvent used to dissolve the drug. After incubation for the indicated time, the medium was replaced by the MTT solution (dissolved in warm PBS, 0.5 mg/mL) and incubated for an additional 3–4 h. The MTT solution was then removed, and DMSO was added to dissolve the blue formazan crystals. Aliquots of each sample were transferred to a 96-well plate, and the absorbance was measured at 570 nm using a microplate reader (Infinite F50) (Tecan, Männedorf, Switzerland). In each experiment, each sample, including the control, had quadruplicate wells to calculate the mean value, which was normalized against the control. The data were presented as the percentage of cell proliferation.

### 4.7. Cell Cycle Analysis by Flow Cytometry

Both flupirtine-treated cells and cells that were serum deprived (0% FBS; serum was later added to reach 10% FBS) were harvested at the indicated time point by trypsinization, washed with ice-cold PBS, and pelleted. The cells were re-suspended in ice-cold PBS, and 1 × 10^6^ cells were fixed with ice-cold ethanol (Sigma Aldrich, St. Louis, MO, USA) at a final concentration of 70% for at least 2 h at −20 °C. The fixed cells were centrifuged, washed with ice-cold PBS, and re-suspended in PBS containing 50 μg/mL RNase A solution (Amresco, Solon, OH, USA). After incubation at 37 °C for 30 min, samples were treated with propidium iodide (PI) at a final concentration of 40 μg/mL and kept cold in the dark. To assess the cell-cycle profiles, the stained samples were measured using a BD FACSCalibur cytometer (BD Bioscience, San Diego, CA, USA) and analyzed using Cell Quest software (BD Bioscience, San Diego, CA, USA).

### 4.8. Western Blot Analysis

The harvested cells were kept on ice while lysed with 1× Passive Lysis Buffer (Promega, Madison, WI, USA) supplemented with a protein-inhibitor cocktail (Sigma Aldrich, St. Louis, MO, USA). Then, the cell debris was pelleted and discarded using a high speed refrigerated centrifuge VS-15000 CFN II (Vision Scientific, Daejeon, Korea), and the proteins were quantified using a BCA protein assay kit (Pierce, Rockford, IL, USA). Thirty micrograms of the protein was denatured in sample buffer by boiling for 5 min at 95 °C, separated using 10% sodium dodecyl sulfate polyacrylamide gel electrophoresis (SDS-PAGE), and transferred to a nitrocellulose transfer membrane (Whatman GmbH, Dassel, Germany). The membranes were blocked in Tris buffered saline-Tween 20 (TBST) containing 5% nonfat milk (5% TBST) (Difco, Sparks, MD, USA) for 1 h at room temperature and incubated overnight at 4 °C with primary antibodies directed against Kv7.5, Sp1, or actin (used as an internal standard) diluted in 5% TBST. On the second day, the membranes were washed three times in TBST and then probed with the secondary antibody for 1 h at room temperature. After being washed three times with TBST, the reactive proteins were visualized using a chemiluminescent detection reagent WesternBright ECL (Advansta, Menlo Park, CA, USA) and scanned with the Vilber Lourmat imaging system (Fusion SL, Marne-la-Vallée, France). Densitometric analysis of the protein bands was performed using the publicly available ImageJ 1.48a software (National Institutes of Health, Bethesda, MD, USA).

### 4.9. Chromatin Immunoprecipitation (ChIP) Assay

The ChIP assay was performed using a kit from Active Motif (Carlsbad, CA, USA) following the manufacturer’s instructions. The cells were seeded into a 15-cm culture plate and incubated overnight until they grew to 70%–80% confluency. After the cells were fixed using formaldehyde, they were scraped and lysed using a Dounce homogenizer (Wheaton, Millville, NJ, USA). The fixed chromatin was enzymatically digested, and a fraction of it was set aside for the examination of chromatin (input) to be used as a positive control in the RT-PCR process.

Immunoprecipitation was performed by incubating the sheared chromatin with an antibody against Sp1 or a nonspecific IgG antibody overnight at 4 °C with end-to-end rotation. To decrease the high background noise, the magnetic beads were blocked with BSA (Millipore; Kankakee, IL, USA). The chromatin-antibody complexes were washed and eluted, leaving only chromatin behind. After treatment with proteinase, the chromatin and the input were ready for PCR.

RT-PCR was used to amplify the sequence spanning the −1197 to −1004 bp region of the *KCNQ5* promoter using the following primers: forward, 5′-GTCGCCAGAGTGTCAGAGGT-3′ and reverse, 5′-AACTGTTAAGCGTCGGCAAT-3′. The final 194 bp PCR product was electrophoresed, as described above.

### 4.10. Statistical Analysis

The values are presented as the mean ± SE in the figures. Significance was analyzed using Student’s *t* test, and the significance levels were set at *p* < 0.05 and 0.01.

## 5. Conclusions

Here, for the first time, we identified Kv7.5 in cancer cells and evaluated its role in canine osteosarcoma cells. We also found that Kv7.5 is upregulated by serum deprivation and that the activation of Kv7.5 by flupirtine leads to cell cycle arrest. In conclusion, this study shows that (i) Kv7.5 may be a useful therapeutic target in the treatment of canine osteosarcoma; and that (ii) the transcription factor Sp1 may play an important role in modulating Kv7.5 expression. Because the Kv7 family is a newly emerging target in pharmacology, and because Kv7.5 is the latest family member to be analyzed, further studies are required to evaluate Kv7.5 and its roles in various stages of cancer, including metastasis, proliferation, and angiogenesis.

## Supplementary Information



## Figures and Tables

**Table 1.** PCR primers for RT-PCR and qPCR.

Gene	Sequences	Product size	Accession number
Kv7.2	F 5′-CCATTGGTTATGGGGACAAG-3′ R 5′-ATAGAACCTCCAGGCCGACT-3′	212	JN546558.1
Kv7.3	F 5′-GCTTCAGCATCTCCCAAGAC-3′ R 5′-GGGAGGGGTCCATACTGAAT-3′	188	XM_532334.3
Kv7.4	F 5′-TGGCCAAAAGGAAATTCAAG-3′ R 5′-CCCCTTGTCTCCCTTCTCTC-3′	179	XM_539568.3
Kv7.5	F 5′-CGCTTTCGTTTTTCTCCTTG-3′ R 5′-GCAGACCAGATCCGAATGAT-3′	156	XM_003431766.1
Sp1	F 5′-TGCAGCAGAATTGAGTCACC-3′ R 5′-CACAACATACTGCCCACCAG-3′	246	XM_543633.3
GAPDH	F 5′-AAGGTCATCCCTGAGCTGAA-3′ R 5′-GACCACCTGGTCCTCAGTGT-3′	192	NM_001003142.1

## References

[1] Lee S.Y., Maniak P.J., Ingbar D.H., O’Grady S.M. (2003). Adult alveolar epithelial cells express multiple subtypes of voltage-gated K^+^ channels that are located in apical membrane. Am. J. Physiol. Cell Physiol.

[2] Vallejo-Gracia A., Bielanska J., Hernandez-Losa J., Castellvi J., Ruiz-Marcellan M.C., Ramony Cajal S., Condom E., Manils J., Soler C., Comes N. (2013). Emerging role for the voltage-dependent K^+^ channel Kv1.5 in B-lymphocyte physiology: Expression associated with human lymphoma malignancy. J. Leukoc. Biol.

[3] Villalonga N., David M., Bielanska J., Vicente R., Comes N., Valenzuela C., Felipe A. (2010). Immunomodulation of voltage-dependent K^+^ channels in macrophages: Molecular and biophysical consequences. J. Gen. Physiol.

[4] Kim H.J., Jang S.H., Jeong Y.A., Ryu P.D., Kim D.Y., Lee S.Y. (2010). Involvement of Kv4.1 K^+^ channels in gastric cancer cell proliferation. Biol. Pharm. Bull.

[5] Suzuki T., Takimoto K. (2004). Selective expression of HERG and Kv2 channels influences proliferation of uterine cancer cells. Int. J. Oncol.

[6] Abdul M., Santo A., Hoosein N. (2003). Activity of potassium channel-blockers in breast cancer. Anticancer Res.

[7] Cherubini A., Taddei G.L., Crociani O., Paglierani M., Buccoliero A.M., Fontana L., Noci I., Borri P., Borrani E., Giachi M. (2000). HERG potassium channels are more frequently expressed in human endometrial cancer as compared to non-cancerous endometrium. Br. J. Cancer.

[8] Jang S.H., Choi C., Hong S.G., Yarishkin O.V., Bae Y.M., Kim J.G., O’Grady S.M., Yoon K.A., Kang K.S., Ryu P.D. (2009). Silencing of Kv4.1 potassium channels inhibits cell proliferation of tumorigenic human mammary epithelial cells. Biochem. Biophys. Res. Commun.

[9] Jang S.H., Kang K.S., Ryu P.D., Lee S.Y. (2009). Kv1.3 voltage-gated K^+^ channel subunit as a potential diagnostic marker and therapeutic target for breast cancer. BMB Rep.

[10] Crociani O., Guasti L., Balzi M., Becchetti A., Wanke E., Olivotto M., Wymore R.S., Arcangeli A. (2003). Cell cycle-dependent expression of HERG1 and HERG1B isoforms in tumor cells. J. Biol. Chem.

[11] Czarnecki A., Dufy-Barbe L., Huet S., Odessa M.F., Bresson-Bepoldin L. (2003). Potassium channel expression level is dependent on the proliferation state in the GH3 pituitary cell line. Am. J. Physiol. Cell Physiol.

[12] Bielanska J., Hernandez-Losa J., Moline T., Somoza R., Cajal S.R., Condom E., Ferreres J.C., Felipe A. (2012). Increased voltage-dependent K^+^ channel Kv1.3 and Kv1.5 expression correlates with leiomyosarcoma aggressiveness. Oncol. Lett.

[13] Brown D.A., Adams P.R. (1980). Muscarinic suppression of a novel voltage-sensitive K^+^ current in a vertebrate neurone. Nature.

[14] Biervert C., Schroeder B.C., Kubisch C., Berkovic S.F., Propping P., Jentsch T.J., Steinlein O.K. (1998). A potassium channel mutation in neonatal human epilepsy. Science.

[15] Maljevic S., Wuttke T.V., Seebohm G., Lerche H. (2010). KV7 channelopathies. Pflugers Arch.

[16] Soldovieri M.V., Miceli F., Taglialatela M. (2011). Driving with no brakes: Molecular pathophysiology of Kv7 potassium channels. Physiology.

[17] Roura-Ferrer M., Sole L., Martinez-Marmol R., Villalonga N., Felipe A. (2008). Skeletal muscle Kv7 (KCNQ) channels in myoblast differentiation and proliferation. Biochem. Biophys. Res. Commun.

[18] Ipavec V., Martire M., Barrese V., Taglialatela M., Curro D. (2011). Kv7 channels regulate muscle tone and nonadrenergic noncholinergic relaxation of the rat gastric fundus. Pharmacol. Res.

[19] Brueggemann L.I., Kakad P.P., Love R.B., Solway J., Dowell M.L., Cribbs L.L., Byron K.L. (2012). Kv7 potassium channels in airway smooth muscle cells: Signal transduction intermediates and pharmacological targets for bronchodilator therapy. Am. J. Physiol. Lung Cell. Mol. Physiol.

[20] Svalo J., Bille M., Parameswaran Theepakaran N., Sheykhzade M., Nordling J., Bouchelouche P. (2013). Bladder contractility is modulated by Kv7 channels in pig detrusor. Eur. J. Pharmacol.

[21] Mani B.K., O’Dowd J., Kumar L., Brueggemann L.I., Ross M., Byron K.L., Vascular K.C.N.Q. (2013). (Kv7) potassium channels as common signaling intermediates and therapeutic targets in cerebral vasospasm. J. Cardiovasc. Pharmacol.

[22] Iannotti F.A., Panza E., Barrese V., Viggiano D., Soldovieri M.V., Taglialatela M. (2010). Expression, localization, and pharmacological role of Kv7 potassium channels in skeletal muscle proliferation, differentiation, and survival after myotoxic insults. J. Pharmacol. Exp. Ther.

[23] Dynan W.S., Tjian R. (1983). The promoter-specific transcription factor Sp1 binds to upstream sequences in the SV40 early promoter. Cell.

[24] Kadonaga J.T., Courey A.J., Ladika J., Tjian R. (1988). Distinct regions of Sp1 modulate DNA binding and transcriptional activation. Science.

[25] Chang W.C., Hung J.J. (2012). Functional role of post-translational modifications of Sp1 in tumorigenesis. J. Biomed. Sci.

[26] Jensen H.S., Callo K., Jespersen T., Jensen B.S., Olesen S.P. (2005). The KCNQ5 potassium channel from mouse: A broadly expressed M-current like potassium channel modulated by zinc, pH, and volume changes. Brain Res. Mol. Brain Res.

[27] Blume S.W., Snyder R.C., Ray R., Thomas S., Koller C.A., Miller D.M. (1991). Mithramycin inhibits SP1 binding and selectively inhibits transcriptional activity of the dihydrofolate reductase gene *in vitro* and *in vivo*. J. Clin. Investig..

[28] Yuan P., Wang L., Wei D., Zhang J., Jia Z., Li Q., Le X., Wang H., Yao J., Xie K. (2007). Therapeutic inhibition of Sp1 expression in growing tumors by mithramycin a correlates directly with potent antiangiogenic effects on human pancreatic cancer. Cancer.

[29] Goodchild C.S., Nelson J., Cooke I., Ashby M., Jackson K. (2008). Combination therapy with flupirtine and opioid: Open-label case series in the treatment of neuropathic pain associated with cancer. Pain Med.

[30] Friedel H.A., Fitton A. (1993). Flupirtine. A review of its pharmacological properties, and therapeutic efficacy in pain states. Drugs.

[31] Burgmaier G., Schonrock L.M., Kuhlmann T., Richter-Landsberg C., Bruck W. (2000). Association of increased bcl-2 expression with rescue from tumor necrosis factor-alpha-induced cell death in the oligodendrocyte cell line OLN-93. J. Neurochem.

[32] Wood J.P., Pergande G., Osborne N.N. (1998). Prevention of glutathione depletion-induced apoptosis in cultured human RPE cells by flupirtine. Restor. Neurol. Neurosci.

[33] Davis P.K., Ho A., Dowdy S.F. (2001). Biological methods for cell-cycle synchronization of mammalian cells. BioTechniques.

[34] Wang Z. (2004). Roles of K^+^ channels in regulating tumour cell proliferation and apoptosis. Pflugers Arch.

[35] Spitzner M., Ousingsawat J., Scheidt K., Kunzelmann K., Schreiber R. (2007). Voltage-gated K^+^ channels support proliferation of colonic carcinoma cells. FASEB J.

[36] Jang S.H., Choi S.Y., Ryu P.D., Lee S.Y. (2011). Anti-proliferative effect of Kv1.3 blockers in A549 human lung adenocarcinoma *in vitro* and *in vivo*. Eur. J. Pharmacol..

[37] Jehle J., Schweizer P.A., Katus H.A., Thomas D. (2011). Novel roles for hERG K^+^ channels in cell proliferation and apoptosis. Cell Death Dis.

[38] Glassmeier G., Hempel K., Wulfsen I., Bauer C.K., Schumacher U., Schwarz J.R. (2012). Inhibition of HERG1 K^+^ channel protein expression decreases cell proliferation of human small cell lung cancer cells. Pflugers Arch.

[39] Asher V., Warren A., Shaw R., Sowter H., Bali A., Khan R. (2011). The role of Eag and HERG channels in cell proliferation and apoptotic cell death in SK-OV-3 ovarian cancer cell line. Cancer Cell Int.

[40] Lee Y.S., Sayeed M.M., Wurster R.D. (1994). *In vitro* antitumor activity of cromakalim in human brain tumor cells. Pharmacology.

[41] Iannotti F.A., Barrese V., Formisano L., Miceli F., Taglialatela M. (2013). Specification of skeletal muscle differentiation by repressor element-1 silencing transcription factor (REST)-regulated Kv7.4 potassium channels. Mol. Biol. Cell.

[42] Mucha M., Ooi L., Linley J.E., Mordaka P., Dalle C., Robertson B., Gamper N., Wood I.C. (2010). Transcriptional control of KCNQ channel genes and the regulation of neuronal excitability. J. Neurosci.

[43] Yang G., Pei Y., Teng H., Cao Q., Wang R. (2011). Specificity protein-1 as a critical regulator of human cystathionine gamma-lyase in smooth muscle cells. J. Biol. Chem.

[44] Ming L., Sakaida T., Yue W., Jha A., Zhang L., Yu J. (2008). Sp1 and p73 activate PUMA following serum starvation. Carcinogenesis.

[45] Vivar O.I., Lin C.L., Firestone G.L., Bjeldanes L.F. (2009). 3,3′-Diindolylmethane induces a G_1_ arrest in human prostate cancer cells irrespective of androgen receptor and p53 status. Biochem. Pharmacol.

[46] Firestone G.L., Bjeldanes L.F. (2003). Indole-3-carbinol and 3-3′-diindolylmethane antiproliferative signaling pathways control cell-cycle gene transcription in human breast cancer cells by regulating promoter-Sp1 transcription factor interactions. J. Nutr.

[47] Beardsley A., Fang K., Mertz H., Castranova V., Friend S., Liu J. (2005). Loss of caveolin-1 polarity impedes endothelial cell polarization and directional movement. J. Biol. Chem.

