# Supplementary material for: Serum Starvation-Induced Voltage-Gated Potassium Channel Kv7.5 Expression and Its Regulation by Sp1 in Canine Osteosarcoma Cells
Source: Int J Mol Sci. 2014 Jan 10;15(1):977–93. doi: 10.3390/ijms15010977 (PMC3907850; doi:10.3390/ijms15010977)
Supplement: Supplementary file 1 [file ijms-15-00977-s001.pdf]

Supplementary Information

Figure S1. CCL-183 cells were arrested in the G_0/G_1 phase and re-entered the S phase following serum deprivation (0% FBS) and re-addition (10% FBS). (**A**) Cell-cycle analysis with propidium iodide was performed using flow cytometry. The representative histograms show cells arrested in the G_0/G_1 phase following serum deprivation (0, −10, −30, −44, −54, and −68 h) and cells that progressed into the S phase following serum re-addition (+14, +24, and +38 h); (**B**) The relative percentage distribution of the G_0/G_1 and S phase was depicted using cells counted from flow cytometry in graphical form. Values are the mean ± SEM of four independent flow cytometry assays. ***** $p < 0.05$ *vs.* 0 h; a, $p < 0.01$ *vs.* −44 h; b, $p < 0.01$ *vs.* −68 h; c, $p < 0.05$ *vs.* −54 h; d, $p < 0.05$ *vs.* −68 h.

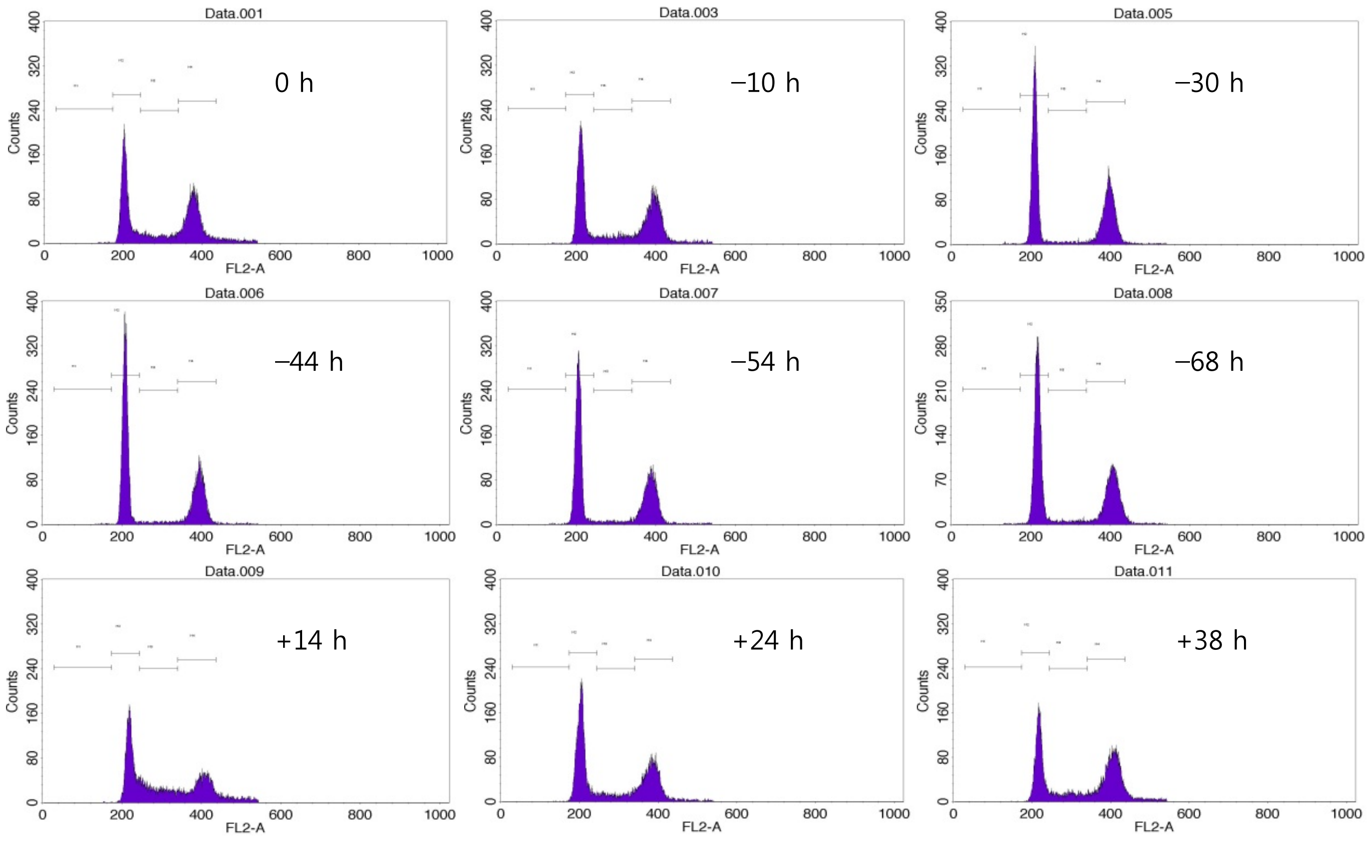

(**A**)

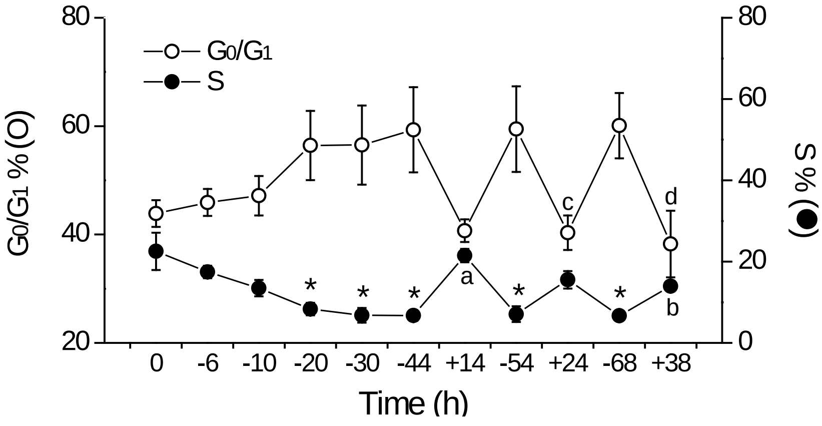

(**B**)
